# A Retrospective Study of the Temporal Trends in Mortality in Patients With Non-alcoholic Fatty Liver Disease and Liver Cirrhosis in the United States Using the Centers for Disease Control and Prevention's (CDC) Multiple Causes of Death (MCD) Database

**DOI:** 10.7759/cureus.90861

**Published:** 2025-08-24

**Authors:** Vinutna Muvva Kolluri, Aayushi Bhamat, Chinmay Gohel, Gowri Bindu, Deepthi Enumula

**Affiliations:** 1 Medicine, All American Institute of Medical Sciences, Black River, JAM; 2 Internal Medicine, Godrej Memorial Hospital, Mumbai, IND; 3 Internal Medicine, Shri Meghji Pethraj Shah Government Medical College, Jamnagar, IND; 4 Internal Medicine, SRM Hospital and Research Center, Kattankulathur, IND; 5 Pharmacy Practice, Balaji Institute of Pharmaceutical Sciences, Warangal, IND; 6 Pharmacy Practice, Sri Ramachandra Institute of Higher Education and Research, Chennai, IND

**Keywords:** age-adjusted mortality rate, cdc mcd, liver cirrhosis, nafld, retrospective study

## Abstract

Introduction

Non-alcoholic fatty liver disease (NAFLD) is a major cause of mortality and its association with liver cirrhosis remains underexplored. Understanding this relationship is essential to identifying high-risk populations and developing targeted public health interventions.

Aims and objective

To analyze mortality trends and demographic disparities in NAFLD with liver cirrhosis as a contributing cause using the Centres for Disease Control and Prevention's (CDC) Wide-Ranging Online Data for Epidemiologic Research (WONDER) Multiple Causes of Death (MCD) database from 1999 to 2020.

Methodology

A retrospective cross-sectional study was conducted using the CDC MCD database to assess mortality trends in individuals aged 25 years and older in the United States from 1999 to 2020. The study included deaths where NAFLD (ICD-10: K76.0) was listed as the underlying cause and liver cirrhosis (ICD-10: K74) as a contributing cause. Data were analyzed by age, gender, race, geographic region, and place of death. Age-adjusted mortality rates (AAMR) and annual percentage change (APC) were calculated.

Results

A total of 14,383 deaths were recorded. The AAMR for NAFLD with liver cirrhosis showed an increase from 1999 to 2006 with an APC of +20.66 (p<0.05), which further showed an increase between 2006 and 2009 with an APC of +27.60, and then again increased significantly from 2009 to 2020 with an APC of 15.87 (p<0.05). The highest mortality was observed in females, the white population, and metropolitan areas. Temporal trends showed an increase, with disparities noted across demographic and geographic factors.

Conclusion

This study highlights significant mortality trends (increasing) in NAFLD with liver cirrhosis, with disparities by gender, race, and location. The findings underscore the need for targeted prevention strategies and improved healthcare access.

## Introduction

Non-alcoholic fatty liver disease (NAFLD), also known as the metabolic dysfunction-associated steatotic liver disease (MASLD), is characterized by the build-up of fat in the liver due to causes other than alcohol consumption. It is the leading cause of chronic liver disease globally [1]. NAFLD can be classified into two broad groups: non-alcoholic fatty liver (NAFL) is defined as the presence of >5% of hepatic steatosis (HS) in the absence of competing liver disease etiologies; it is associated with a lower risk of progression to further liver damage and complications [1]; the other being non-alcoholic steatohepatitis (NASH), which is defined histologically by the presence of HS with evidence for hepatocyte damage along with a higher risk of progression to fibrosis and further cirrhosis [2].

The worldwide incidence of NAFLD is about 25%, with its occurrence ranging over 80 million in the United States alone, and its occurrence and progression increase with age. Men seem to have a greater overall incidence of NAFL, whereas women seem to have a slightly higher prevalence of NASH with more severe stages of fibrosis. Numerous risk factors can be attributed to NAFLD, including metabolic comorbidities such as obesity, metabolic syndrome, type 2 diabetes mellitus, dyslipidemia, and insulin resistance, along with other genetic and environmental factors [3]. The most common cause of death among NAFLD patients is cardiovascular diseases, followed by extrahepatic complications and hepatic complications [4].

Liver cirrhosis is characterized by diffuse hepatic fibrosis with replacement of the normal hepatic anatomy with regenerative hepatic nodules, most commonly due to inflammation and long-standing liver damage [5]. Liver cirrhosis can be broadly classified into compensated or uncompensated cirrhosis based on the ability of the liver to perform the basic functions despite the structural damage. The most common risk factors that can be attributed to liver cirrhosis include consumption of alcohol, NASH, type 2 diabetes mellitus, chronic infections such as hepatitis B or C virus, autoimmune causes (autoimmune hepatitis, primary biliary cholangitis), genetic causes, and also other external contributing factors, which include prolonged exposure to certain medications or chemicals (methotrexate) [6]. It is prevalent worldwide owing to a high morbidity and mortality rate. The mortality risk is five times higher for individuals with compensated cirrhosis and ten times higher for those with decompensated cirrhosis than for the general population [7].

Approximately 20% of individuals with NAFLD are expected to develop NASH, and within this group, around 20% may advance to cirrhosis over the course of three to four decades [8]. The most common clinical presentation of NAFLD cirrhosis includes a woman in the age group of 40-50 years with known comorbidities such as obesity and/or type 2 diabetes mellitus, and the most common clinical feature they present with is ascites [9].

The aim and objective of this study is to assess mortality in patients with NAFLD where liver cirrhosis is a contributing cause of death, using the Centre for Disease Control and Prevention's (CDC) Wide-Ranging Online Data for Epidemiologic Research (WONDER) Multiple Causes of Death (MCD) database. This study analyzes mortality trends from 1999 to 2020 and classifies data by sex, race, and geographic location to identify discrepancies in mortality patterns.

## Materials and methods

A retrospective cross-sectional study was conducted with the help of the CDC WONDER MCD database [10]. Publicly available data on mortality comprising de-identified death certificate information for all deaths recorded in the United States was used for the purpose of this study. The study is exempt from institutional review board (IRB) approval [11] because the dataset that was employed consists of publicly available, de-identified information. Hence, the study was classified as non-human participant research.

Mortality data were extracted from the CDC WONDER MCD database for the years 1999-2020. NAFLD-related deaths are uncommon in populations under the age of 25. Therefore, only adults aged 25 or above were considered for the study. NAFLD (ICD-10: K76.0) was selected as the underlying cause of death, while liver cirrhosis (ICD-10: K74) was selected as the multiple cause of death to assess the co-occurrence of these conditions. To analyze disparities in mortality outcomes, individuals of various demographics, such as gender (male and female) and race/ethnicity (American Indian or Alaska Native, Asian or Pacific Islander, Black or African American, or White), were included. Geographic variables included urbanization based on the 2013 classification, categorizing metropolitan cities into large central metro, large fringe metro, medium metro, and small metro, and non-metropolitan cities into micropolitan and non-core rural areas. Furthermore, the place of death was classified into four categories: medical facility, home, hospice, or nursing facility. Mortality rates were standardized using age-adjusted rates per 1,000,000 population, with adjustments based on the U.S. Standard Population from the year 2000 to allow for accurate comparisons over time.

To encapsulate both demographic and geographic variables, descriptive statistics along with numbers and percentages were utilized. With the help of the CDC WONDER MCD database, age-adjusted mortality rates (AAMRs) were calculated for each subgroup. JoinPoint Regression Analysis (JoinPoint Software Version 5.3.0.0, November 2024) was not only employed to evaluate temporal trends but also used to determine annual percentage changes (APC) in NAFLD-related mortality with cirrhosis of the liver as a contributing cause. Trends were assessed over the 1999-2020 study period to identify statistically significant changes in mortality patterns across different demographic and geographic groups.

## Results

In the years 1999-2020, the CDC MCD database recorded 14,383 deaths in the United States among individuals aged 25 years and older. Among these, deaths where NAFLD (ICD-10: K76.0) was listed as the underlying cause of death and liver cirrhosis (ICD-10: K74) was recorded as a multiple cause of death were included in the study (14,383). The crude mortality rate for NAFLD with liver cirrhosis as a contributing cause was 3.2 per 1,000,000 population. Deaths due to causes other than these criteria were excluded.

Demographic characteristics

Among the total deaths analyzed, women accounted for 9159 (63.7%) cases, while men accounted for 5224 (36.3%) cases. The mortality rate for NAFLD with liver cirrhosis as a contributing cause was higher in females compared to males, indicating a potential demographic disparity.

Regarding racial distribution, the highest proportion of deaths occurred among White individuals (n=13,640, 94.8%), followed by Black or African American individuals (n=295, 2.1%), Asian or Pacific Islander individuals (n=219, 1.5%), and American Indian or Alaska Native individuals (n=229, 1.6%). The mortality burden was highest among white individuals, highlighting racial disparities in mortality trends related to NAFLD and liver cirrhosis.

Geographic characteristics

A majority of deaths occurred in metropolitan areas (n=11,190, 77.8%), while non-metropolitan areas accounted for 3193 (22.2%) deaths. Regarding the place of death, most deaths occurred in medical facilities (n=6477, 45.03%), followed by decedents' homes (n=4065, 28.26%), nursing homes, or long-term care facilities (n=1683, 11.70%), and hospice facilities (n=1644, 11.43%).

Temporal trends

**Figure 1:**
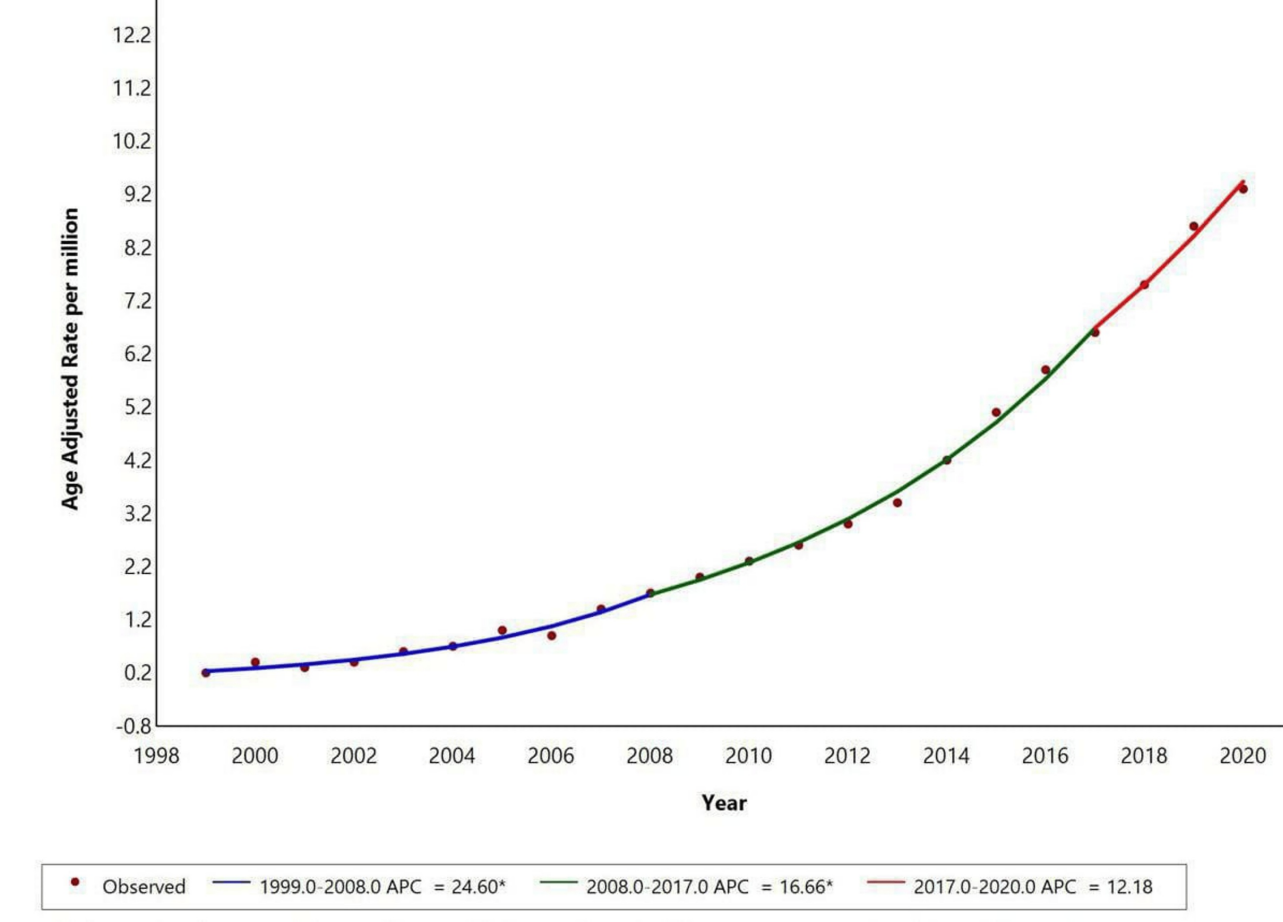
Overall age-adjusted mortality rates among adults in the United States, 1999–2020. * Indicates that the annual percent change (APC) is significantly different from zero at the alpha=0.05 level. Final Selected Model: 1 Joinpoint. Figure created using Joinpoint software.

When stratified by gender, women showed a significant increase in the trend from 1999 to 2008 with an APC of +24.6 (p<0.05) and then from 2008 to 2017 with an APC of +16.66 (p<0.05). It showed a further increase in trend from 2017 to 2020 with an APC of +12.18. Temporal trends for men were not displayed due to data suppression for counts <10, limiting reliable trend analysis as represented in Figure 1.

**Figure 2:**
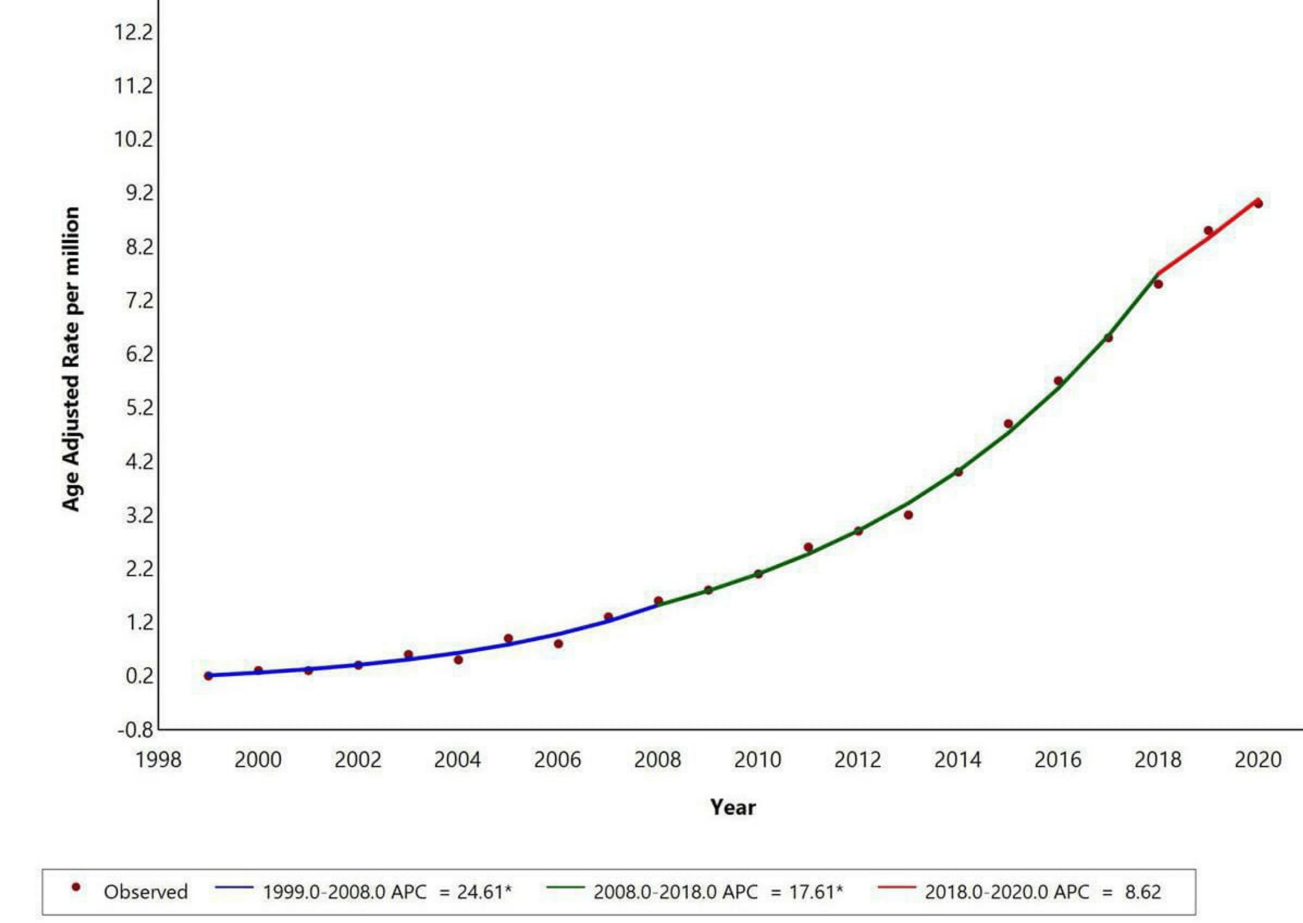
Trends in age-adjusted mortality rates among adults in the United States from 1999-2020 based on gender * Indicates that the annual percent change (APC) is significantly different from zero at the alpha=0.05 level. Final selected model: 1 Joinpoint. Figure created using Joinpoint software.

Racial disparities were observed in mortality trends. White individuals showed a significant increase in the trend from 1999 to 2008 with an APC of +24.61 (p<0.05) and then from 2008 to 2018 with an APC of +17.61 (p<0.05). It showed a further increase in trend from 2018 to 2020 with an APC of +8.62. Temporal trends for American Indian/Alaska Native, Asian Pacific Islander, and Black or African American populations are not displayed due to data suppression for counts <10, limiting reliable trend analysis as shown in Figure 2.

**Figure 3:**
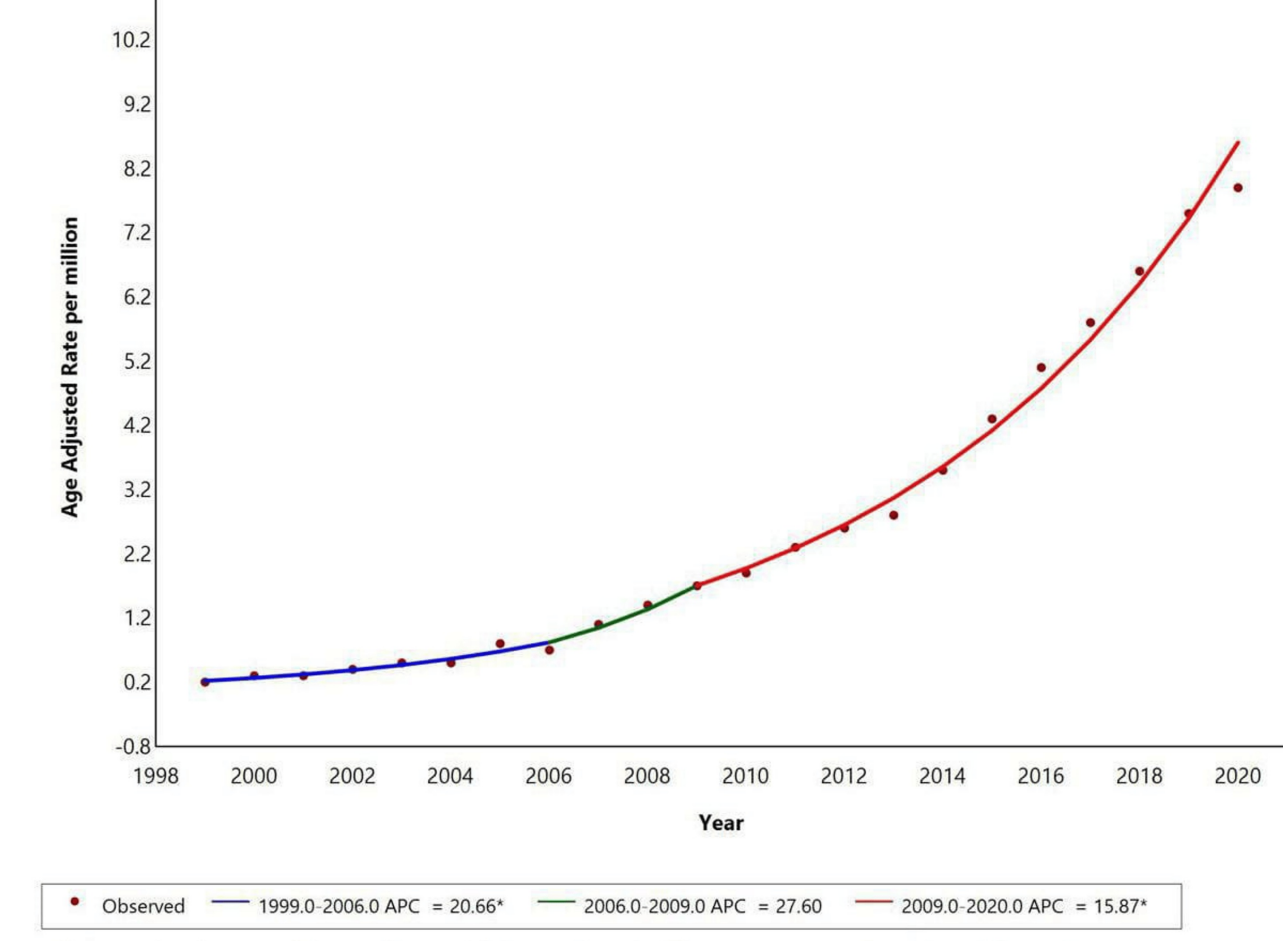
Trends in age-adjusted mortality rates stratified by race among adults in the United States, 1999 to 2020. * Indicates that the annual percent change (APC) is significantly different from zero at the alpha=0.05 level. Final selected model: 1 Joinpoint. Figure created using Joinpoint software.

From 1999 to 2020, AAMR for NAFLD with liver cirrhosis as a contributing cause showed an increase. The AAMR showed a significant increase from 1999 to 2006 with an APC of +20.66 (p<0.05), which further showed an increase between 2006 and 2009 with an APC of +27.60 and then again increased significantly from 2009 to 2020 with an APC of 15.87 (p<0.05), as shown in Figure 3.

## Discussion

A retrospective study utilizing the CDC MCD database examined mortality trends related to NAFLD (ICD: K76) with liver cirrhosis (ICD: K74) as a contributing cause among individuals aged 25 and older in the United States from 1999 to 2020. This study identified a total of 14,383 deaths.

During the study period from 1999 to 2020, AAMR for NAFLD with liver cirrhosis as a contributing cause increased continuously, with significant APC of +24.60% between 1999 and 2008, +16.66% between 2008 and 2017, and +12.18% between 2017 and 2020 (p<0.05 for all). Mortality was highest among women (63.7%), White individuals (94.8%), those residing in metropolitan areas (77.8%), and deaths occurring in medical facilities (45.03%).

NAFLD encompasses a range of liver disorders marked by excessive fat buildup in the liver, independent of alcohol consumption, which vary from simple steatosis to non-alcoholic steatohepatitis (NASH), which can advance to fibrosis, cirrhosis, and even hepatocellular carcinoma (HCC) [12]. Risk factors for NAFLD progression include insulin resistance, hypertension, genetic susceptibility, and dyslipidemia [13]. Ultrasound and MRI biomarkers are useful for diagnosing NAFLD. MRI proton density fat fraction (MRI-PDFF) is more effective than liver biopsy, especially for tracking long-term changes in liver fat. The stage of fibrosis is most strongly correlated with liver decompensation and mortality, with elastography emerging as a dependable biomarker for assessing liver fibrosis [14]. Serum ANGPTL8 levels could serve as a potential and specific diagnostic marker for liver fibrosis, and targeting ANGPTL8 shows significant promise in the development of novel therapies for treating NAFLD-related liver fibrosis [15]. Hispanic ethnicity and genetic polymorphisms in PNPLA3, TM6SF2, GCKR, MBOAT7, and HSD17B13 are associated with steatosis-related lipotoxicity and oxidative DNA damage, which can contribute to hepatocarcinogenesis. These factors may help explain the link between NAFLD and HCC, particularly in cases where cirrhosis is absent [16].

This study found that NAFLD with liver cirrhosis had an overall 14,383 deaths with a 3.2 per million crude rate and APC (1999-2006: 20.6; 2006-2009: 27.6; 2009-2020: 15.87). Currently, there are no validated models that integrate multiple risk factors and fibrosis stages into "HCC risk calculators" for NAFLD patients. Developing such tools would allow for better risk stratification, help identify high-risk patients even without cirrhosis, and enable personalized, risk-based surveillance strategies [16]. Over the past 20 years, the incidence of non-viral NAFLD/NASH-related HCC has risen sharply. Since there are no effective drugs available for treating NAFLD and NASH, a class of thiazolidinediones (TZDs), typically used to manage type 2 diabetes, is occasionally employed to improve liver function despite the potential for side effects [17]. Chronic liver disease (CLD), including cirrhosis and liver cancer, significantly impacts mortality, morbidity, and economics.

The study found that women had a higher AAMR compared to males, while white individuals were at a greater risk than other ethnic groups. Additionally, individuals living in metropolitan areas faced an increased risk of mortality, and a higher number of deaths occurred in medical facilities. Cirrhosis is rising in high Socio-Demographic Index (SDI) regions, while HCC rates are increasing in the wealthiest countries, with the global CLD burden growing unevenly across sociodemographic factors [1]. NAFLD affects a quarter of the global population, with NASH incidence expected to rise by 56% in the next decade. It is the fastest-growing cause of HCC in countries like the United States, France, and the UK, with the prevalence set to increase alongside the obesity epidemic. Urgent action on metabolic risk factors and awareness is needed, as emerging evidence suggests immune dysfunction, gut inflammation, and dysbiosis play key roles in NAFLD-related HCC [18].

AAMR was significantly higher in metropolitan areas at 77.8%, compared to 22.2% in non-metropolitan areas. This suggests that urban residents may face greater health risks or have less access to healthcare, influencing higher mortality in these regions. NAFLD susceptibility is influenced by environmental factors like diet and exercise, as well as genetic, epigenetic, and microbiota-related factors. Nutritional genomics, which includes nutrigenetics and nutri-epigenomics, explores how diet interacts with the genome to affect health, offering potential insights for improving clinical and nutrition practices [19]. NAFLD, including its progressive form NASH, is a leading cause of liver transplant but lacks Food and Drug Administration (FDA)-approved therapies. The goal is to optimize future therapeutic strategies by exploring lifestyle changes, pharmacological agents, surgical approaches, and the gut microbiome in NASH treatment, focusing on insulin sensitizers, thyroid hormone mimetics, antioxidants, and other potential targets, thereby lowering the mortality rates [20].

Between 1999 and 2020, AAMR for NAFLD with liver cirrhosis as a contributing cause increased significantly, with substantial rises from 1999 to 2006 (APC: 20.66), 2006 to 2009 (APC: 27.60), and 2009 to 2020 (APC: 15.87). In women, the AAMR showed a significant increase from 1999 to 2008 (APC: 24.6), followed by a slower rise from 2008 to 2017 (APC: 16.66), and a continued upward trend from 2017 to 2020 (APC: 12.18). Data for men was unavailable due to suppression of counts under 10. Racial disparities were observed, with White individuals showing significant increases from 1999 to 2008 (APC: 24.61), 2008 to 2018 (APC: 17.61), and a continued rise from 2018 to 2020 (APC: 8.62). Data for other racial groups were suppressed due to low counts. Metabolic dysfunction-associated steatotic liver disease (MASLD), previously known as NAFLD, is characterized by liver fat accumulation in the presence of cardiometabolic risk factors, without harmful alcohol intake. MASLD can progress to metabolic dysfunction-associated steatohepatitis (MASH), fibrosis, cirrhosis, and MASH-related HCC [21].

The observed mortality trends highlight the need for further research on integrating multiple risk factors for screening NAFLD and developing screening tools and managing accordingly to reduce the risk of mortality. Screening for MASLD should focus on individuals with cardiometabolic risk factors, abnormal liver enzymes, or hepatic steatosis signs, especially those with type 2 diabetes or obesity. A stepwise approach using blood tests (like fibrosis-4 (FIB-4) index) and imaging techniques (like transient elastography) helps identify advanced fibrosis. Treatment includes lifestyle modifications, management of comorbidities, and the use of incretin-based therapies for type 2 diabetes mellitus and obesity. Bariatric surgery is an option for obese patients, and MASH-targeted treatments, like resmetirom, are recommended for non-cirrhotic MASH with significant fibrosis. For cirrhosis, management includes metabolic drugs, nutritional counselling, and monitoring for portal hypertension and HCC, with liver transplantation considered for decompensated cirrhosis according to most recent studies [21].

Limitations

Mortality assessment for NAFLD with liver cirrhosis faces several limitations, including data suppression for small populations, particularly among certain racial groups, which limits accurate trend analysis. AAMR may not adequately account for the complex interactions of comorbidities like obesity and diabetes, which affect outcomes. Misclassification of NAFLD-related deaths and inconsistent diagnostic criteria further undermine data reliability. The lack of consistent gender- and race-specific data also limits the understanding of demographic disparities in mortality trends. Additionally, the absence of standardized reporting across regions complicates cross-regional comparisons and makes generalizing findings more difficult. The use of ICD-10 code K76.0, the only available code for NAFLD in the CDC WONDER database, may lead to misclassification and underestimation of the true burden.

## Conclusions

This study highlights significant trends in mortality for NAFLD with liver cirrhosis, showing an increase in AAMR from 1999 to 2009, followed by a decrease from 2009 to 2020. It also identifies notable disparities in mortality based on gender, race, urbanization, and place of death. Women and White individuals exhibited higher mortality rates over time, with urban areas experiencing a heavier mortality burden than rural areas. Additionally, the majority of deaths occurred in medical facilities, suggesting potential gaps in access to care, especially in non-metropolitan regions. These findings underline the importance of developing targeted prevention and intervention strategies for at-risk groups, such as women, racial minorities, and urban populations. Enhancing healthcare access and addressing the root causes of these disparities are critical in mitigating the increasing mortality associated with NAFLD-related liver cirrhosis.

## References

[1] Younossi ZM, Koenig AB, Abdelatif D, Fazel Y, Henry L, Wymer M (2016). Global epidemiology of nonalcoholic fatty liver disease - meta-analytic assessment of prevalence, incidence, and outcomes. Hepatology.

[2] Chalasani N, Younossi Z, Lavine JE (2012). The diagnosis and management of non-alcoholic fatty liver disease: practice Guideline by the American Association for the Study of Liver Diseases, American College of Gastroenterology, and the American Gastroenterological Association. Hepatology.

[3] Cotter TG, Rinella M (2020). Nonalcoholic fatty liver disease 2020: the state of the disease. Gastroenterology.

[4] Byrne CD, Targher G (2015). NAFLD: a multisystem disease. J Hepatol.

[5] Campana L, Esser H, Huch M, Forbes S (2021). Liver regeneration and inflammation: from fundamental science to clinical applications. Nat Rev Molec Cell Biol.

[6] Cirrhosis in Over 16s: assessment and management (2023). NICE Clinical Guidelines No. 50.

[7] Devarbhavi H, Asrani SK, Arab JP, Nartey YA, Pose E, Kamath PS (2023). Global burden of liver disease: 2023 update. J Hepatol.

[8] Singh S, Allen AM, Wang Z, Prokop LJ, Murad MH, Loomba R (2015). Fibrosis progression in nonalcoholic fatty liver vs nonalcoholic steatohepatitis: a systematic review and meta-analysis of paired-biopsy studies. Clin Gastroenterol Hepatol.

[9] Li B, Zhang C, Zhan YT (2018). Nonalcoholic fatty liver disease cirrhosis: a review of its epidemiology, risk factors, clinical presentation, diagnosis, management, and prognosis. Can J Gastroenterol Hepatol.

[10] ( 1999). Centers for Disease Control and Prevention (CDC), National Center for Health Statistics. CDC WONDER Online Database, Multiple Cause of Death Files. http://wonder.cdc.gov.

[11] De Lusignan S, Liyanage H, Di Iorio CT, Chan T, Liaw ST (2015). Using routinely collected health data for surveillance, quality improvement and research: Framework and key questions to assess ethics and privacy and enable data access. BMJ Health & Care Informatics.

[12] Saiman Y, Duarte-Rojo A, Rinella ME (2022). Fatty liver disease: diagnosis and stratification. Annu Rev Med.

[13] Pouwels S, Sakran N, Graham Y (2022). Non-alcoholic fatty liver disease (NAFLD): a review of pathophysiology, clinical management and effects of weight loss. BMC Endocr Disord.

[14] Ajmera V, Loomba R (2021). Imaging biomarkers of NAFLD, NASH, and fibrosis. Mol Metab.

[15] Zhang Z, Yuan Y, Hu L (2023). ANGPTL8 accelerates liver fibrosis mediated by HFD-induced inflammatory activity via LILRB2/ERK signaling pathways. J Adv Res.

[16] Ioannou GN (2021). Epidemiology and risk-stratification of NAFLD-associated HCC. J Hepatol.

[17] Kim H, Lee DS, An TH, Park HJ, Kim WK, Bae KH, Oh KJ (2021). Metabolic spectrum of liver failure in type 2 diabetes and obesity: from NAFLD to NASH to HCC. Int J Mol Sci.

[18] Huang DQ, El-Serag HB, Loomba R (2021). Global epidemiology of NAFLD-related HCC: trends, predictions, risk factors and prevention. Nat Rev Gastroenterol Hepatol.

[19] Meroni M, Longo M, Rustichelli A, Dongiovanni P (2020). Nutrition and genetics in NAFLD: the perfect binomium. Int J Mol Sci.

[20] Raza S, Rajak S, Upadhyay A, Tewari A, Anthony Sinha R (2021). Current treatment paradigms and emerging therapies for NAFLD/NASH. Front Biosci (Landmark Ed).

[21] (2024). EASL-EASD-EASO Clinical Practice Guidelines on the management of metabolic dysfunction-associated steatotic liver disease (MASLD). J Hepatol.

